# Pseudohypoadrenalism, a subclinical cortisol metabolism disorder in hyperuricemia

**DOI:** 10.3389/fendo.2023.1279205

**Published:** 2023-11-16

**Authors:** Ruixia Bao, Beibei Chen, Jujie Pan, Alexander Wang, Haiyang Yu, Qian Chen, Yi Zhang, Tao Wang

**Affiliations:** ^1^ State Key Laboratory of Component-based Chinese Medicine, Tianjin University of Traditional Chinese Medicine, Jinghai District, Tianjin, China; ^2^ College of Education, University of Texas at Austin, Austin, TX, United States; ^3^ State Key Laboratory of Bioactive Substance and Function of Natural Medicines, Institute of Materia Medica, Chinese Academy of Medical Sciences and Peking Union Medical College, Beijing, China

**Keywords:** hyperuricemia, liver injury, lipid metabolism disorder, cortisol, pseudohypoadrenalism

## Abstract

**Background:**

Hyperuricemia is a known risk factor of lipid metabolism disorder. However, the mechanisms have not been fully understood.

**Methods:**

The serum samples from hyperuricemia subjects were used to analyze the correlation between serum uric acid and clinical characteristics. Hyperuricemia mice induced by potassium oxonate (PO) and adenine were used to explore glucocorticoid metabolism.

**Results:**

In hyperuricemia patients, the levels of serum uric acid were positively correlated with the levels of γ-glutamyltransferase, associated with a cortisol metabolism disorder. In hyperuricemia state, the adrenal glands failed to respond to adrenocorticotropic hormone properly, leading to low cortisol, but not corticosterone production, and decreased mRNA levels of aldosterone synthase, 11β-hydroxylase, and 3β-hydroxysteroid dehydrogenase 1, three key enzymes for cortisol synthesis. The expression of both hepatic 5α-reductase and renal 11β-hydroxysteroid dehydrogenase 2 was significantly reduced, which led to low cortisol clearance. We denominated this cortisol metabolism disorder in hyperuricemia as pseudohypoadrenalism (PHAL).

**Conclusion:**

PHAL increased exposure to the bioavailable cortisol in the liver, leading to local amplification of the biological action of corticosteroids. Unregulated biosynthesis pathway of bile acid expanded bile acid pool, and further aggravated cholestatic liver injury.

## Graphical Abstract

Associated with hyperuricemia, liver injury induced imbalanced cortisol metabolism with the characteristics include suppressed HPA axis, decreased cortisol production and secretion, impaired cortisol clearance, and higher bioactive cortisol level.

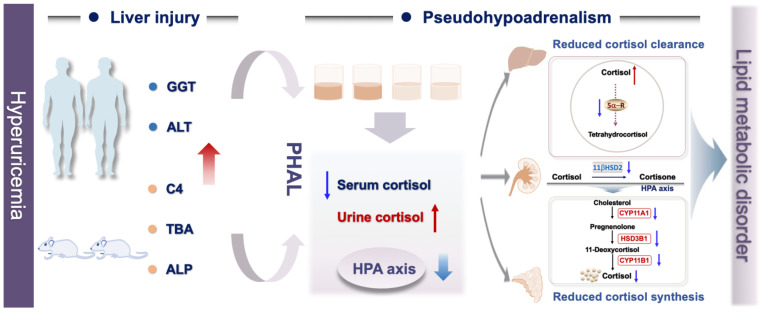

## 1 Introduction

It is well established that hyperuricemia is strongly associated with depression, lipid metabolism disorder, hypertension, and atherosclerosis (1). However, the pathological mechanism between them is unclear. Our group analyzed the serum samples of hyperuricemia patients, and found that serum uric acid levels were positively correlated with γ-glutamyltransferase (GGT), a biomarker of damage to the hepatic cells or bile ducts (2).

A variety of factors could elevate serum GGT level. Extrinsic factors include medications, viruses, and mechanical damage (3), while intrinsic factors are reported as imbalanced hormones, bile acids, and amino acids (4). Cortisol is a glucocorticoid hormone secreted by the adrenal cortex, modulating the biosynthesis of lipids, regulating stress response and blood pressure, controlling sleep-wake cycle. It has been reported that over-exposure to intrinsic glucocorticoids led to cholestasis, liver injury, and cardiovascular disease (5 **;** 6). Our group found higher levels of free cortisol in the hyperuricemia mice. We hypothesized that hyperuricemia caused abnormal metabolism of cortisol, which could be one of the causes of hyperuricemia related hepatic injury and other hyperuricemia complications.

In this study, we found that hyperuricemia suppressed hypothalamic–pituitary–adrenal (HPA) axis, and decreased cortisol production and secretion. Meanwhile, due to impaired renal and hepatic functions caused by hyperuricemia, the cortisol clearance decreased, which led to higher cumulative exposure to bioavailable cortisol in the hyperuricemia patients compared to that in the control group. We denominated this abnormal metabolism of cortisol as “pseudohypoadrenalism (PHAL)”, which might be one of the causes of hyperuricemia related diseases, such as depression, hyperlipidemia and cardiovascular disease.

## 2 Materials and methods

### 2.1 Reagents

Potassium oxonate (PO), adenine, and dexamethasone were purchased from Sigma-Aldrich (St. Louis, MO). Adrenocorticotropic Hormone (ACTH) Fragment 1-24 human was purchased from Yuanye Biotechnology Co., Ltd. (Shanghai, China). Cortisol assay kit (Human and Mouse), GGT assay kit (Mouse) and ACTH assay kit (mouse) were purchased from AmyJet Scientific Co., Ltd (Wuhan, China).

### 2.2 Population of study

Participants were men who had undergone healthy examinations in the Second teaching hospital of Tianjin University of Traditional Chinese Medicine. All participants gave written informed consent, and the study was approved by local ethics committees (No. 2021-020-01).

#### 2.2.1 Inclusion criteria

Male.

Participants 18 ≤ age ≤60 years at the time informed consent was obtained.

Participants were diagnosed with hyperuricemia.

#### 2.2.2 Exclusion criteria

Secondary hyperuricemia.

Participants with a previous history of organ transplantation or extracorporeal circulation.

Participants with heart, lung, brain, liver, kidney, hematopoietic system and other serious diseases, including hypertension, viral hepatitis B or C, kidney stones, polycystic kidney disease, hematological malignancy or other unconfirmed malignant diseases and HIV.

A total of 422 participants were included according to the inclusion and exclusion criteria.

#### 2.2.3 Serum collection

After overnight fast, serum sample of healthy and hyperuricemia participants were collected under aseptic conditions at 7:00 am to 9:00 am. According to the manufacturer’s instructions, serum cortisol levels were determined with a commercial ELISA kit.

#### 2.2.4 Data collection

Measurement data include age, height, weight, body mass index (BMI), systolic blood pressure (SBP), diastolic blood pressure (DBP) and heart rate. Laboratory indicators include direct bilirubin, GGT, high-density lipoprotein (HDL), low-density lipoprotein (LDL), globulin, aspartate aminotransferase (AST), total protein, albumin, alanine aminotransferase (ALT), total bilirubin, total cholesterol (TC), triglyceride (TG), fasting glucose, blood urea nitrogen (BUN), serum uric acid, creatinine. All clinical biochemical indicators were uniformly determined in the laboratory of the hospital.

### 2.3 Animal

Male C57BL/6J mice, SPF grade, 8 weeks old, were purchased from Beijing Vital River Laboratory Animal Technology Co., Ltd. (Beijing, China). All animals had free access to standard diet and water, and were housed in experimental conditions at 25 ± 2°C with humidity of 60 ± 5% in a fixed 12 h artificial light period. Before the experiments, they were allowed at least 7 days to adapt to their living environment. All animal experiment designs were approved by the Science and Technological Committee and the Animal Use and Care Committee of TJUTCM (TCM-LAEC2022007).

### 2.4 PO and adenine induced hyperuricemia mice

Potassium oxonate was use as uricase inhibitor to block conversion uric acid to allantoin and oral administration of adenine was used to increase purine intake. Mice were randomly divided into the control group and the hyperuricemia group, each group included 8 mice. Hyperuricemia mice were induced by oral administration of adenine (50 mg/kg/day, 20 ml/kg body weight) and PO (200 mg/kg/day, 20 ml/kg body weight) once a day in the morning for 20 consecutive days. The control mice received the same volume of distilled water. At the end of the administration, serum, liver, kidney and adrenal glands were collected under isoflurane anesthesia.

### 2.5 ACTH stimulation test on hyperuricemia mice

Mice were randomly divided into the control group and the hyperuricemia group, each group including 8 mice. Hyperuricemia mice were induced by oral administration of adenine (50 mg/kg/day, 20 ml/kg body weight) and PO (200 mg/kg/day, 20 ml/kg body weight) once a day in the morning for 20 consecutive days. On the 21st day, blood samples at baseline were collected before the control and hyperuricemia were received ACTH Fragment 1-24 human (100 μg/kg body weight) by intraperitoneal injection. And then, mice were decapitated rapidly, blood samples were collected 30 and 60 min after the ACTH administration. According to the manufacturer’s instructions, serum cortisol levels were determined with a commercial ELISA kit.

### 2.6 Dexamethasone suppression test on hyperuricemia mice

C57BL/6J mice were randomly divided into the control group and the hyperuricemia group (n=8). Hyperuricemia mice were induced by oral administration of adenine and PO as previously described in “ACTH stimulation test on hyperuricemia mice”. On the 21st day, the control and hyperuricemia mice were intraperitoneally injected with dexamethasone (0.1 mg/kg body weight), and eight hours later, mice were decapitated rapidly, and the blood samples were collected. According to the manufacturer’s instructions, serum cortisol and ACTH levels were determined with a commercial ELISA kit.

### 2.7 Serum uric acid analysis

Ultra-Performance Liquid Chromatography (UPLC) analysis was used to determine serum uric acid levels in mice as described previously (7). Serum TC, alkaline phosphatase (ALP), GGT, and total bile acid (TBA) were determined with commercial kits according to the manufacturer’s instructions.

### 2.8 Serum 7α-hydroxycholest-4-en-3-one (C4) analysis

Serum C4 levels were determined through UPLC-MS analysis, a serum sample (25 μL) was added into 1 mL of ice-cold acetonitrile and then d7-C4 (10 ng/mL) was added as an internal standard. After vortexed thoroughly for 5 min, the mixture was centrifuged at 14,000 g for 10 minutes under 4 °C. The supernatant was evaporated under nitrogen flow. The residue was re-dissolved in 50 μL of 100% methanol, and centrifuged at 20,000 g for 10 minutes under 4°C. The supernatant was directly used for UHPLC/Q-Orbitrap-MS analysis. Detection and quantification were achieved by Waters UPLC system (Waters Corporation, Milford, MA, USA) coupled with a Exactive™ Plus Orbitrap mass spectrometer equipped with an ESI source (ThermoFisher Scientific, Waltham, MA, USA). Mass spectrometry acquisition mode was parallel reaction monitoring and the collision energy was 30 V. C4 were separated using a Waters ACQUITY UPLC HSS T3 column (2.1 × 100 mm, 1.8 μm) with 10 mM ammonium formate (Sigma-Aldrich (ST. Louis, MO, USA) in methanol. The working solution were prepared at a series concentration of 100 ng/mL, 50 ng/mL, 20 ng/mL, 10 ng/mL, 4 ng/mL, 2 ng/mL, 0.8 ng/mL, 0.4 ng/mL of serum C4. The column temperature was 40°C, the flow rate was 0.2 mL/min, and the injection volume was 5 μl. The data acquisition and analysis were carried out using Xcalibur™ Software 4.0.

### 2.9 Quantitative real time polymerase chain reaction

RNA isolation, cDNA synthesis, and qRT-PCR analysis were performed as described previously (8). The primers used for qRT-PCR were synthesized by Dingguo Bio Co. Ltd, Shanghai, China. Sequences used for qRT-PCR were shown in **Table 1**. Results were presented as levels of expression relative to those of controls after normalization to GAPDH using the 2-^△△CT^ methods.

**Table 1:** Primer Sequences for qRT-PCR.

Gene name	Forward sequence (5’ to3’)	Reverse sequence (5’ to3’)
11βHSD1	TGGTGCTCTTCCTGGCCT	CCCAGTGACAATCACTTTCTTT
11βHSD2	AACCTCTGGCAGAAACGCAAG	GGCATCTACAACTGGGCTAAGG
5α-reductase	AGCCAGTTTGCGGTGTATG	TTCTCAGATTCCGCAGGATG
5β-reductase	GAAAAGATAGCAGAAGGGAAGGT	GGGACATGCTCTGTATTCCATAA
ABCG5	CCCGTTCTGAGCTTTTTCAG	CAAGGGTAACCGCAGTCATT
ABCG8	GACAGCTTCACAGCCCACAA	GCCTGAAGATGTCAGAGCGA
BSEP	CTGCCAAGGATGCTAATGCA	CGATGGCTACCCTTTGCTTCT
CYP11A1	GGTTCCACTCCTCAAAGCCA	GGATCTCGACCCATGGCAAA
CYP11B1	GCCTGAACGCTATATGCCTC	CACGTGGAAGGATTTCAGCAC
CYP11B2	5′-AGCATCGCTGCAAATCCTCA-3′	5′-GGTTTCGGCCCATGGAGTAG-3
CYP17A1	CTGGGCACTGCATCACGATA	GATAAAGAGCTCCTGCCGGG
CYP21A2	CTTTCCTGCTTCACCACCCTG	CCTTGGATGTTGGGGATGATG
CYP27A1	TGCCTGGGTCGGAGGAT	GAGCCAGGGCAATCTCATACTT
CYP7A1	CTGGGCTGTGCTCTGAAGT	GGGAGTTTGTGATGAAGTGGA
CYP7B1	TGAGGTTCTGAGGCTGTGC	TGGAGGAAAGAGGGCTACAA
CYP8B1	ACAGCGTGATGGAGGAGAGT	AGGGGAAGAGAGCCACCTTA
FGF15	TGTTTCACCGCTCCTTCTTT	TCTACATCCTCCACCATCCTG
FGFR4	GCATCTTTCAGGGGACACCA	TTGTACCAGTGACGACCACG
FXR	CCCCTGCTTGATGTGCTAC	CGTGGTGATGGTTGAATGTC
GAPDH	TGTGTCCGTCGTGGATCTGA	CCTGCTTCACCACCTTCTTGAT
GRα	AAAGAGCTAGGAAAAGCCATTGTC	CTGTCTTTGGGCTTTTGAGATAGG
GRβ	AAAGAGCTAGGAAAAGCCATTGTC	TCAGCTAACATCTCTGGGAATTCA
HMGCR	AGCTTGCCCGAATTGTATGTG	TCTGTTGTGAACCATGTGACTTC
HNF4α	AAATGTGCAGGTGTTGACCA	CACGCTCCTCCTGAAGAATC
HSD3B1	5′-TCCACACTGCTGCTGTCATT-3′	5′-AGATGAAGGCTGGCACACTT-3′
LRH	TTGAGTGGGCCAGGAGTAGT	ACGCGACTTCTGTGTGTGAG
NPC1L1	GCAAGGTGATCAGGAGGTTGA	ATCCTCATCCTGGGCTTTGC
NTCP	ATGACCACCTGCTCCAGCTT	GCCTTTGTAGGGCACCTTGT
SHP	CGATCCTCTTCAACCCAGATG	AGGGCTCCAAGACTTCACACA
SREBP2	TGGAAGTGACCGAGAGTCCC	GAGACTGCTCCACAGGTGAC

### 2.10 Hematoxylin and eosin staining on adrenal glands

Adrenal glands were fixed with 4% paraformaldehyde and then embedded in paraffin. The tissues were sectioned into 5 μm-thick and stained with H&E according to a standard protocol, and the treated slices were photographed (magnification, 100×) with Axio Imager D2 (Zeiss, Oberkochen, Germany). The number of cells of zona fasciculata in the adrenal glands was counted using Image J analysis software (Version 1.0, National Institutes of Health, Bethesda, MD, USA).

### 2.11 Statistical analysis

SPSS 20.0 statistical software (version 20, SPSS; IBM, Armonk, NY, USA) was used to perform statistical analysis. Data were expressed as the mean ± S.E.M. Spearman correlation analyses were used to evaluate the correlation between serum uric acid and clinical characteristics. The independent t-test was used to compare the differences between the control mice and the hyperuricemia mice. Significant differences between the means of SUA quartile 1- quartile 4 groups were evaluated by one-way analysis of variance (ANOVA). The LSD and Dunnett’s test were used for *post hoc* evaluations. *p*<0.05 was considered to represent a statistically significant difference.

## 3 Results

### 3.1 Abnormal biomarkers of mild liver injury were tightly linked to hyperuricemia

Spearman correlation analyses were used to determine whether there were associations between serum uric acid level and clinical characteristics. As shown in **Table 2**, the levels of ALT (*r*=0.167, *p*=0.000), creatinine (*r*=0.211, *p*=0.000), GGT (*r*=0.228, *p*=0.000), LDL-C (*r*=0.223, *p*=0.000), TC (*r*=0.223, *p*=0.000) and TG (*r*=0.214, *p*=0.000) were significantly positively correlated with serum uric acid levels in hyperuricemia patients. In addition, demographic characteristics including age, height, and weight were also significantly correlated with serum uric acid levels in the hyperuricemia subjects.

**Table 2:** Correlation between serum uric acid and clinical characteristics in hyperuricemia subjects.

Parameter (standard error)	Overall (n=422)	*R*	*P*
Age (years)	51.21± 10.72	-0.214***	0.000
Height (cm)	172.09± 6.70	0.135**	0.005
Weight (kg)	82.26 + 12.46	0.117*	0.014
B MI (kg/m2)	27.67± 3.42	0.068	0.152
SBP (mm Hg)	134.76± 15.04	0.022	0.641
DBP (mm Hg)	87.92± 11.17	0.099*	0.038
Heart rate (beats/min)	73.05± 8.15	0.004	0.939
Direct Bilirubin (μmol/L)	3.57± 1.56	-0.091	0.058
GGT (U/L)	46.51± 55.74	0.228***	0.000
H DL (mmol/L)	1.26± 0.24	-0.092	0.054
L DL (mmol/L)	3.15± 0.82	0.223***	0.000
Globulin (g/L)	30.00± 3.83	0.09	0.06
A ST (U/L)	25.32± 15.24	0.087	0.067
Total protein (g/L)	73.72± 4.03	0.08	0.094
Albumin (g/L)	43.86± 2.48	-0.019	0.698
A LT (U/L)	32.64± 22.06	0.167***	0.000
Total bilirubin (μmol/L)	14.27± 5.84	-0.058	0.222
T C (mmol/L)	5.24± 1.03	0.234***	0.000
TG (mmol/L)	2.41± 1.46	0.214***	0.000
Fasting glucose (mmol/L)	5.42± 1.58	0.000	0.999
B UN (mmol/L)	4.87± 1.18	0.042	0.378
Creatinine (μmol/L)	74.42± 14.33	0.211***	0.000

Data are presented as mean ± S.D. *P<0.05, **P<0.01, ***P<0.001.

BMI, body mass index; SBP, systolic blood pressure; DBP, diastolic blood pressure; GGT, γ-glutamyltransferase; HDL, high-density lipoprotein; LDL, low-density lipoprotein; AST, aspartate aminotransferase; ALT, alanine aminotransferase; TC, total cholesterol; TG, triglyceride; BUN, blood urea nitrogen.

The slightly elevated serum levels of ALT and GGT indicated a mild to moderate progression of liver cell and duct cell damage (2). The characteristics of individuals according to quartiles of serum uric acid levels are summarized in **Table 3**. The Q1, Q2, Q3 and Q4 quartiles of serum uric acid were 352.89± 52.62, 449.35± 19.62, 509.84 ± 17.76 and 610.09 ± 57.73 μmol/L, respectively. There were no statistically significant differences in BMI, SBP, DBP, HDL-C, AST, total protein, albumin, total bilirubin, fasting glucose, and BUN among the quartiles of serum uric acid. However, it is notable that the GGT and ALT levels were 39.41% and 28.19% higher, respectively, in the Q4 quartile than the counterparts in the Q1 quartile of serum uric acid. These results confirmed that hyperuricemia was associated with higher prevalence of mild liver injury.

**Table 3:** Demographic and clinical characteristics of hyperuricemia subjects according to the quartiles of serum uric acid.

Parameter (standard error)	Q1 (n=105)	Q2 (n=105)	Q3 (n=106)	Q4 (n=106)	*P*
Age (years)	51.66 ± 11.34	53.41 ± 9.55	52.05 ± 9.90	46.70 ± 10.66***	0.000
Height (cm)	172.85 ± 6.21	172.01 ± 6.03	172.03 ± 5.70	173.83 ± 6.39	0.096
Weight (kg)	83.18 ± 11.97	81.43 ± 11.64	81.25± 10.74	86.13 ± 13.36	0.010
B MI (kg/m2)	27.76 ± 3.46	27.44 ± 3.32	27.41 ± 3.39	28.37 ± 3.46	0.138
SBP (mm Hg)	133.69 ± 14.50	134.39 ± 15.45	135.68 ± 15.31	135.46 ± 14.97	0.749
DBP (mm Hg)	86.92 ± 10.02	87.26 ± 11.67	88.63 ± 11.14	89.72 ± 11.24	0.230
Heart rate (beats/min)	72.99 ± 9.09	72.46 ± 7.54	72.79 ± 8.63	73.62 ± 7.09	0.771
Direct Bilirubin (μmol/L)	3.94 ± 1.81	3.63 ± 1.40	3.54 ± 1.75	3.35± 1.18**	0.046
G GT(U/L)	40.04 ± 27.15	43.33 ± 47.74	51.25 ± 88.59	55.82 ± 43.12*	0.161
H DL (mmol/L)	1.26 ± 0.23	1.26 ± 0.27	1.28 ± 0.21	1.23 ± 0.24	0.408
L DL (mmol/L)	2.98 ± 0.83	3.05 ± 0.77	3.22 ± 0.75*	3.41 ± 0.81***	0.000
Globulin (g/L)	29.64 ± 3.39	29.51 ± 4.11	29.73 ± 3.63	30.93± 3.88*	0.022
A ST (U/L)	24.23 ± 11.86	24.24 ± 11.53	26.42 ± 20.55	27.08 ± 16.20	0.411
Total protein (g/L)	73.52 ± 3.63	73.22 ± 4.42	73.50 ± 3.85	74.56 ± 4.13	0.081
Albumin (g/L)	44.12 ± 2.58	43.74 ± 2.75	43.92 ± 2.11	43.78 ± 2.49	0.683
A LT (U/L)	30.37 ± 18.38	30.70 ± 20.13	32.31 ± 23.77	38.93 ± 25.49*	0.017
Total bilirubin (μmol/L)	15.14 ± 5.91	14.15 ± 5.67	14.33 ± 6.62	13.87 ± 5.10	0.431
T C (mmol/L)	4.98 ± 0.99	5.07 ± 0.94	5.36 ± 1.11**	5.56 ± 0.95**	0.000
TG (mmol/L)	2.18 ± 1.27	2.16 ± 1.02	2.38 ± 1.41	2.87 ± 1.75**	0.001
Fasting glucose (mmol/L)	5.80 ± 2.40	5.33 ± 1.37	5.37 ± 1.19	5.18 ± 0.75	0.024
B UN (mmol/L)	4.87 ± 1.07	4.76 ± 1.38	4.84 ± 1.08	5.00 ± 1.16	0.487
Serum uric acid (μmol/L)	352.89± 52.62	449.35± 19.62***	509.84 ± 17.76***	610.09 ± 57.73***	0.000
Creatinine (μmol/L)	73.10 ± 14.06	74.34 ± 13.57	74.23 ± 13.46	79.18 ± 13.89**	0.007

Data are presented as mean ± S.D. *P<0.05, **P<0.01, ***P<0.001 vs Q1.

BMI, body mass index; SBP, systolic blood pressure; DBP, diastolic blood pressure; GGT, γ-glutamyltransferase; HDL, high-density lipoprotein; LDL, low-density lipoprotein; AST, aspartate aminotransferase; ALT, alanine aminotransferase; TC, total cholesterol; TG, triglyceride; BUN, blood urea nitrogen.

### 3.2 Serum levels of C4, ALP, and TBA were elevated in hyperuricemia mice

**Figure 1:**
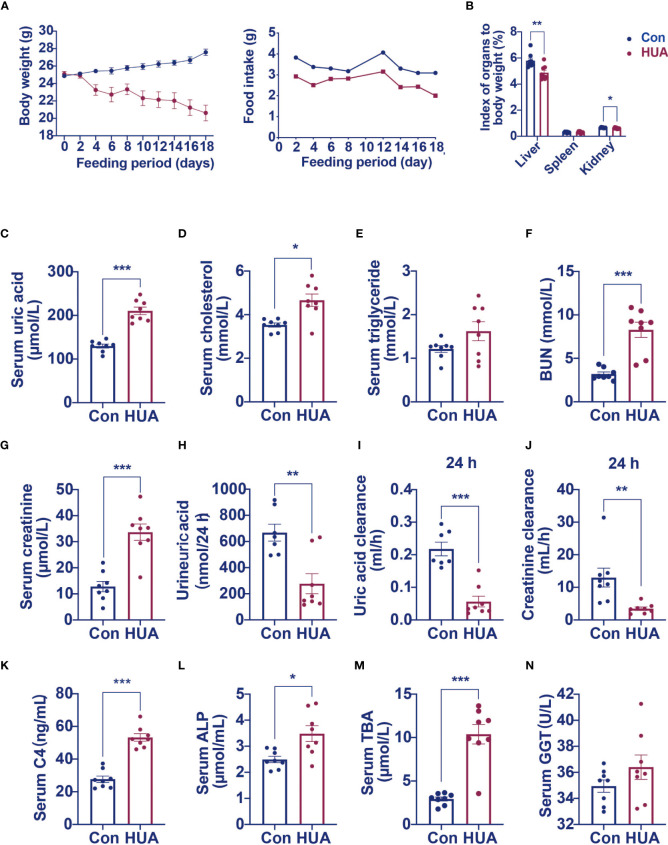
Serum levels of C4, ALP and TBA were elevated in hyperuricemia mice (n=8). Normal control C57BL/6J mice group (Con), PO and adenine induced hyperuricemia mice group (HUA). **(A)** The body weight and food intake of control and hyperuricemia mice. **(B)** The organ index of control and hyperuricemia mice. **(C)** Serum uric acid level. **(D)** Serum cholesterol level. **(E)** Serum triglycerides level. **(F)** Serum BUN level. **(G)** Serum creatinine level. **(H)** Uric acid excretion in 24h urine. **(I)** Uric acid clearance in 24 h. **(J)** Creatinine clearance in 24 h. **(K)** Serum C4 level. **(L)** Serum ALP level. **(M)** Serum TBA level. **(N)** Serum GGT level. Data are presented as mean ± S.E.M. *p < 0.05, **p < 0.01, ***p < 0.001 vs control mice group.

We next examined the effects of hyperuricemia on liver injury in mice. As shown in **Figure 1A**, unlike patients with hyperuricemia, PO and adenine induced hyperuricemia mice have markedly lower body weights compared to the control mice. The liver index and kidney index were significantly lower, with no significant difference in spleen index in the hyperuricemia mice when compared to those in the controls (**Figure 1B**). As expected, the serum uric acid level of the hyperuricemia group was 62% higher compared with the control (**Figure 1C**). Meanwhile, the serum TC level was higher in the hyperuricemia group compared with the control (**Figure 1D**), consistent with the clinical characteristics of hyperuricemia patients. The serum TG level was slightly elevated in the hyperuricemia mice more than the control mice (**Figure 1E**). The serum BUN and creatinine levels were significantly higher in the hyperuricemia mice, 160% more than the control mice (**Figures 1F, G**), similarly, in hyperuricemia patients, there is a significant positive correlation between serum uric acid and creatinine levels. Urine uric acid levels and 24 h clearances of uric acid and creatinine were significantly lower in the hyperuricemia group in comparison to the control group (**Figures 1H–J**).

High level of GGT was considered as a biomarker of liver or bile duct damage. We measured C4 (a marker of bile acid synthesis), ALP (a marker of cholestasis), and TBA levels to identify changes in bile acid metabolism in the hyperuricemia mice. We found significantly higher serum C4 (92% higher), ALP (40% higher), and TBA (254% higher) levels in the hyperuricemia group when compared to the control group (**Figures 1K–M**). GGT level was only slightly elevated in the hyperuricemia mice **Figure 1N**, however, there is a significant positive correlation between blood uric acid and GGT in hyperuricemia patients. These data indicated that hyperuricemia led to dysregulation of bile acid metabolism, which might partly contribute to the liver cell damage.

### 3.3 The expression patterns of 7α-hydroxylase (CYP7A1) and other bile acid metabolism genes in hyperuricemia induced liver injury was similar to that of cholestasis

**Figure 2:**
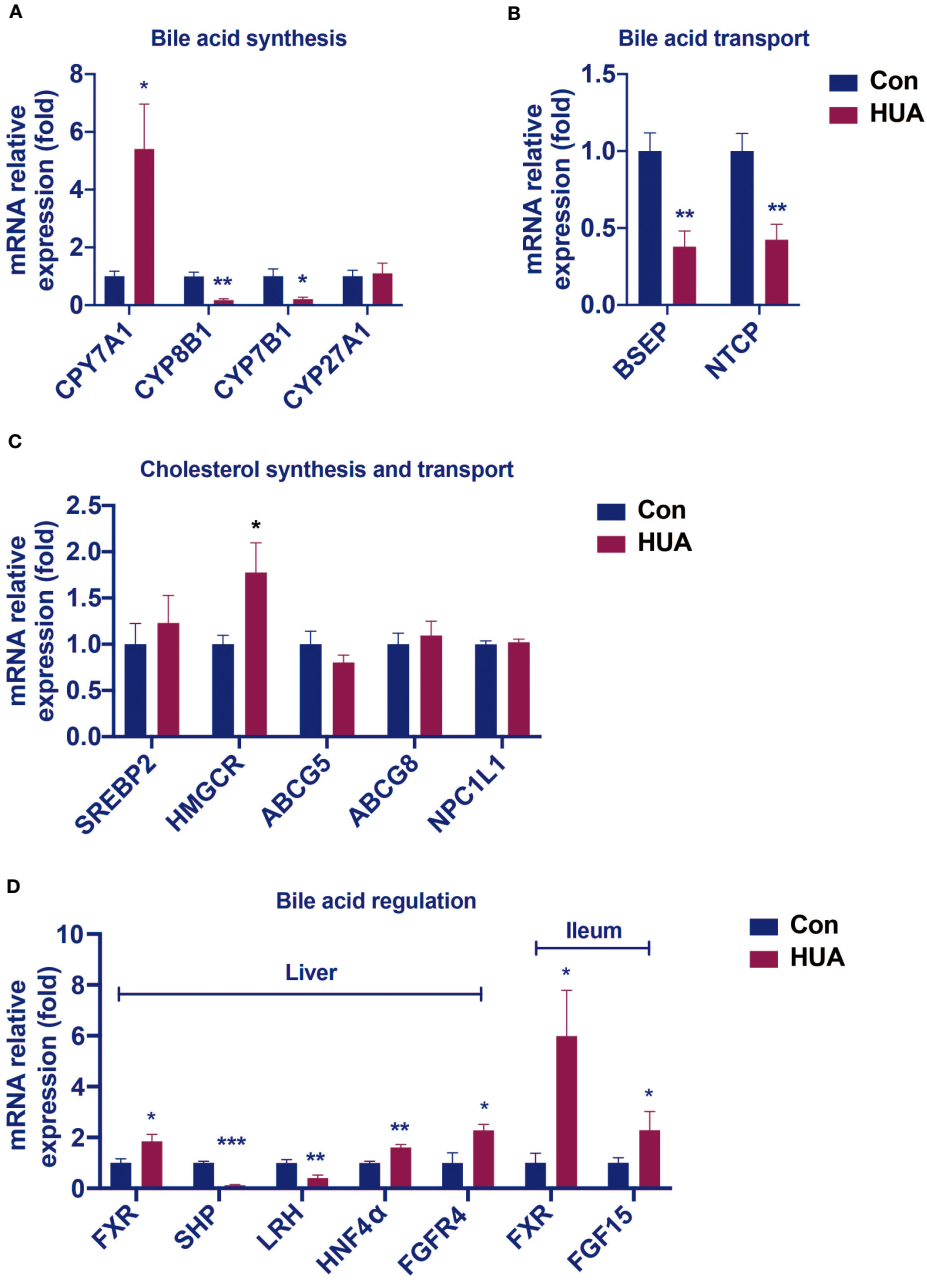
Changes in the hepatic mRNA levels of genes involved in BA and cholesterol homeostasis in hyperuricemia mice. Normal control C57BL/6J mice group (Con), PO and adenine induced hyperuricemia mice group (HUA). **(A)** The mRNA levels of genes involved in bile acid synthesis. **(B)** The mRNA levels of genes involved in bile acid transport. **(C)** The mRNA levels of genes involved in cholesterol synthesis and transport. **(D)** The mRNA levels of genes involved in bile acid regulation. Data are presented as mean ± S.E.M. ^*^ *p* < 0.05, ^**^ *p* < 0.01, ^***^ *p* < 0.001 vs control mice group.

Hyperuricemia mice showed elevated levels of ALP, TBA, and C4, which are similar to the early changes in cholestasis (9). We further tested the expression of cholesterol synthesis genes, bile acid synthesis genes, and bile acid transport genes in hyperuricemia mice. Compared with the control group, the mRNA levels of cholesterol synthesis regulatory enzyme 3-hydroxy-3-methylglutaryl-CoA reductase (HMGCR) was 77% higher (**Figure 2C**), and transcription factors hepatocyte nuclear factor 4α (HNF4α) 60% higher, whereas the mRNA levels of transcription factors small heterodimer partner (SHP) and liver receptor homolog (LRH) (**Figure 2D**) were 88% and 60% lower, respectively, and bile acid transporters bile salt export pump (BSEP) and sodium taurocholate co-transporting polypeptide (NTCP) 62% and 57% lower, respectively (**Figure 2B**).

In the liver of the hyperuricemia mice, CYP7A1 expression (**Figure 2A**) was significantly higher than the control by 441%. These data indicated that hyperuricemia related impairment of bile acid homeostasis was similar to cholestasis.

### 3.4 Cortisol stimulated hepatic secretion of fibroblast growth factor 21 played an important role in CYP7A1 over-expression in hyperuricemia mice

Compared with the control group, the hyperuricemia mice showed increased mRNA levels of hepatic FXR and FGFR4, as well as the ileal FXR and FGF15 (**Figure 2D**), which were different from those in cholestasis.

**Figure 3:**
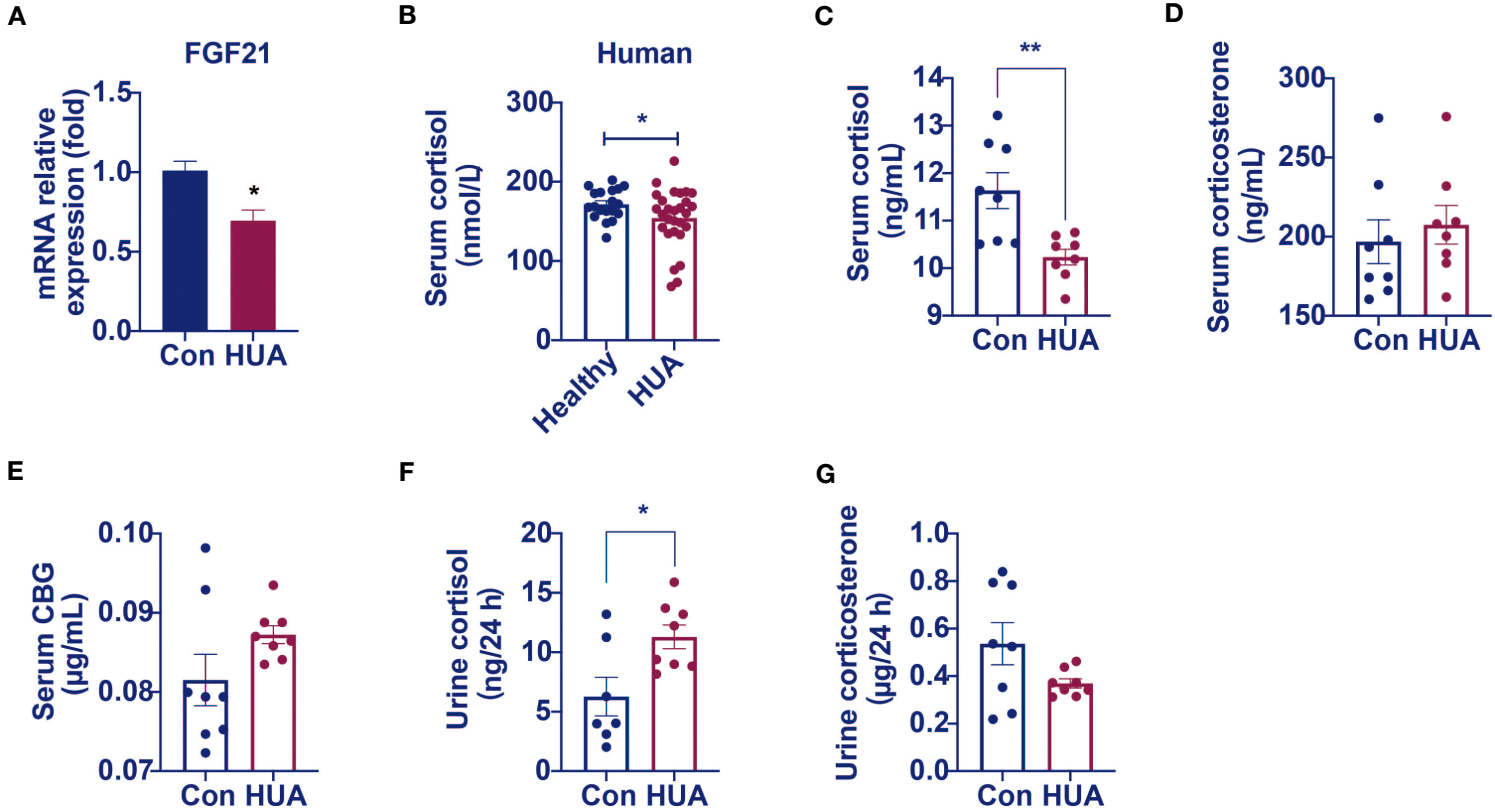
Circulating cortisol levels decreased and 24 h urine free cortisol levels increased in hyperuricemia mice. Normal control C57BL/6J mice group (Con), PO and adenine induced hyperuricemia mice group (HUA). **(A)** The mRNA level of hepatic FGF21. **(B)** Serum cortisol level in hyperuricemia subjects. **(C)** Serum cortisol level in control and hyperuricemia mice. **(D)** Serum corticosterone level in control and hyperuricemia mice. **(E)** Serum CBG level in control and hyperuricemia mice. **(F)** 24 h urine free cortisol in control and hyperuricemia mice. **(G)** 24 h urine corticosterone in control and hyperuricemia mice. Data are presented as mean ± S.E.M. ^*^ *p* < 0.05, ^**^ *p* < 0.01, ^***^ *p* < 0.001 vs control mice group.

We found that the relative mRNA levels of hepatic FGF21 was lower by 31% in the hyperuricemia mice than that in the control (**Figure 3A**), demonstrating that the over-expression of CYP7A1 in the liver was linked to a glucocorticoid disorder. Further studies revealed that serum cortisol level was lower (**Figures 3B, C**) both in hyperuricemia human and hyperuricemia mice, and 24-h urine cortisol was higher (**Figure 3E**) in hyperuricemia mice with normal corticosteroid-binding globulin (CBG) level (**Figure 3F**). Serum corticosterone level and 24 h urine corticosterone in hyperuricemia mice showed no significant difference compared with control group (**Figures 3D, G**). The results suggested an abnormal cortisol metabolism pattern occurred in the hyperuricemia subjects. We denominated it as PHAL.

### 3.5 The adrenal glands in hyperuricemia mice showed no obvious pathological damage, but the HPA axis negative feedback loop was inhibited

**Figure 4:**
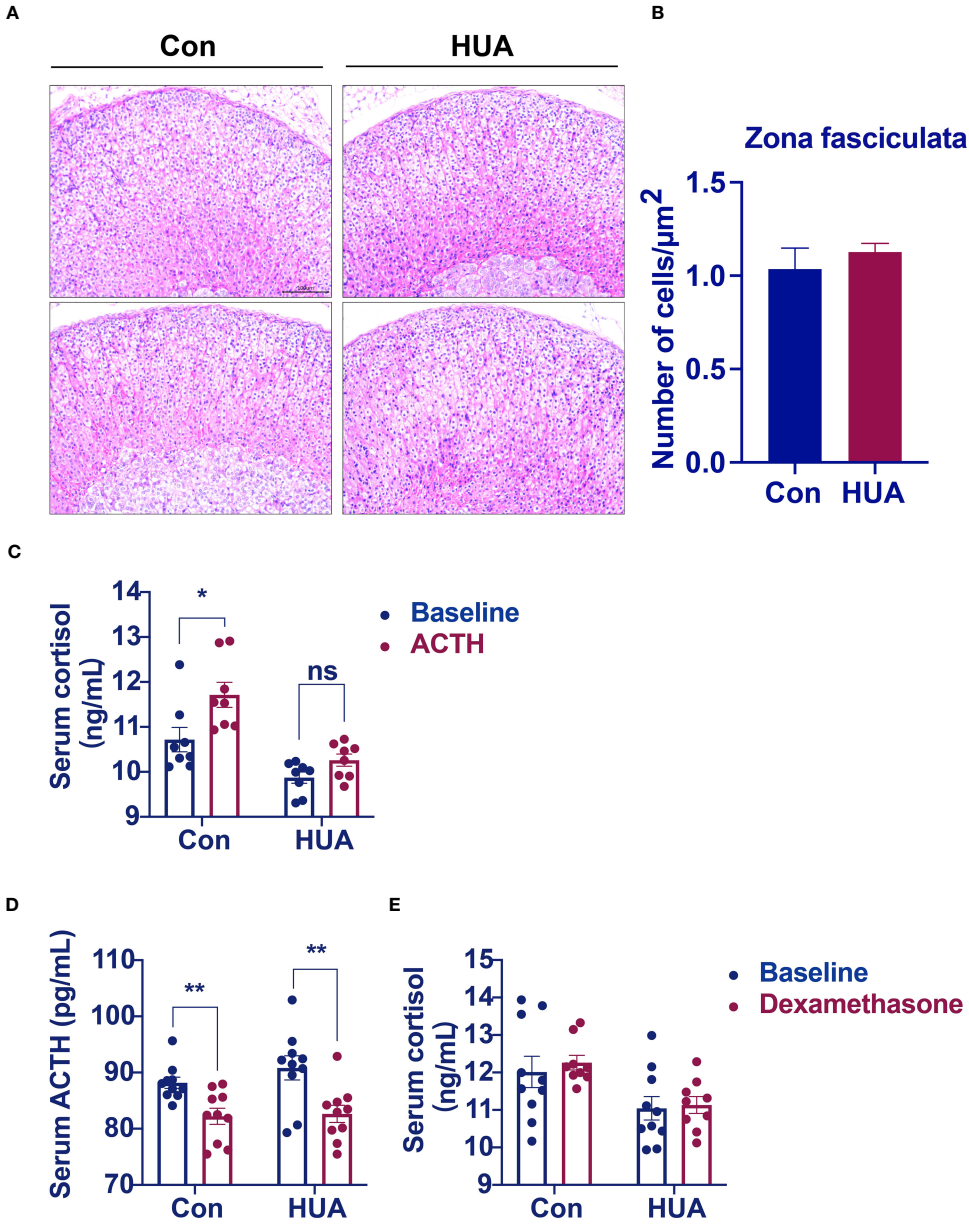
Morphological analysis of the adrenal glands. Normal control C57BL/6J mice group (Con), PO and adenine induced hyperuricemia mice group (HUA). **(A)** Adrenal glands for H&E staining (20x). **(B)** The number of cells zona fasciculata of the adrenal gland. **(C)** Serum cortisol level of mice in ACTH stimulation test. **(D, E)** Serum ACTH and cortisol levels of mice in dexamethasone suppression test. Data are presented as mean ± S.E.M. ns, no significance ^*^ *p* < 0.05, ^**^ *p* < 0.01 vs control mice group.

The cells of the adrenal cortex were arranged in a regular pattern, with rich cytoplasm, round or oval nuclei, and uniform cell size in both the hyperuricemia mice and the control. However, the zona fasciculata cells were enlarged and irregularly arranged in the hyperuricemia mice (**Figure 4A**). The number of cells per unit area was not significantly different between the hyperuricemia mice and the control (**Figure 4B**). Considering the insufficient cortisol levels in the hyperuricemia mice, we performed ACTH stimulation test to examine the adrenocortical function. The serum cortisol levels increased in the control mice after ACTH administration, but not in the hyperuricemia mice, indicating that the adrenal glands of the hyperuricemia mice were not responsive to ACTH (**Figure 4C**). Furthermore, considering the increased 24h urine cortisol, a dexamethasone suppression test was used to evaluate the negative feedback control of the HPA axis. ACTH and cortisol levels were suppressed in the control mice in response to dexamethasone, indicating that the HPA axis was intact (**Figure 4D**). However, in the hyperuricemia mice, although the ACTH level was suppressed, the cortisol level showed no response to dexamethasone (**Figures 4D, E**). Taken together, these data demonstrated that the HPA axis of the hyperuricemia mice was partially resistant to feedback inhibition by exogenous steroids, which is different from those in hypercortisolism and adrenal insufficiency.

### 3.6 PHAL had decreased clearance of cortisol and synthesis of steroidogenic enzyme

**Figure 5:**
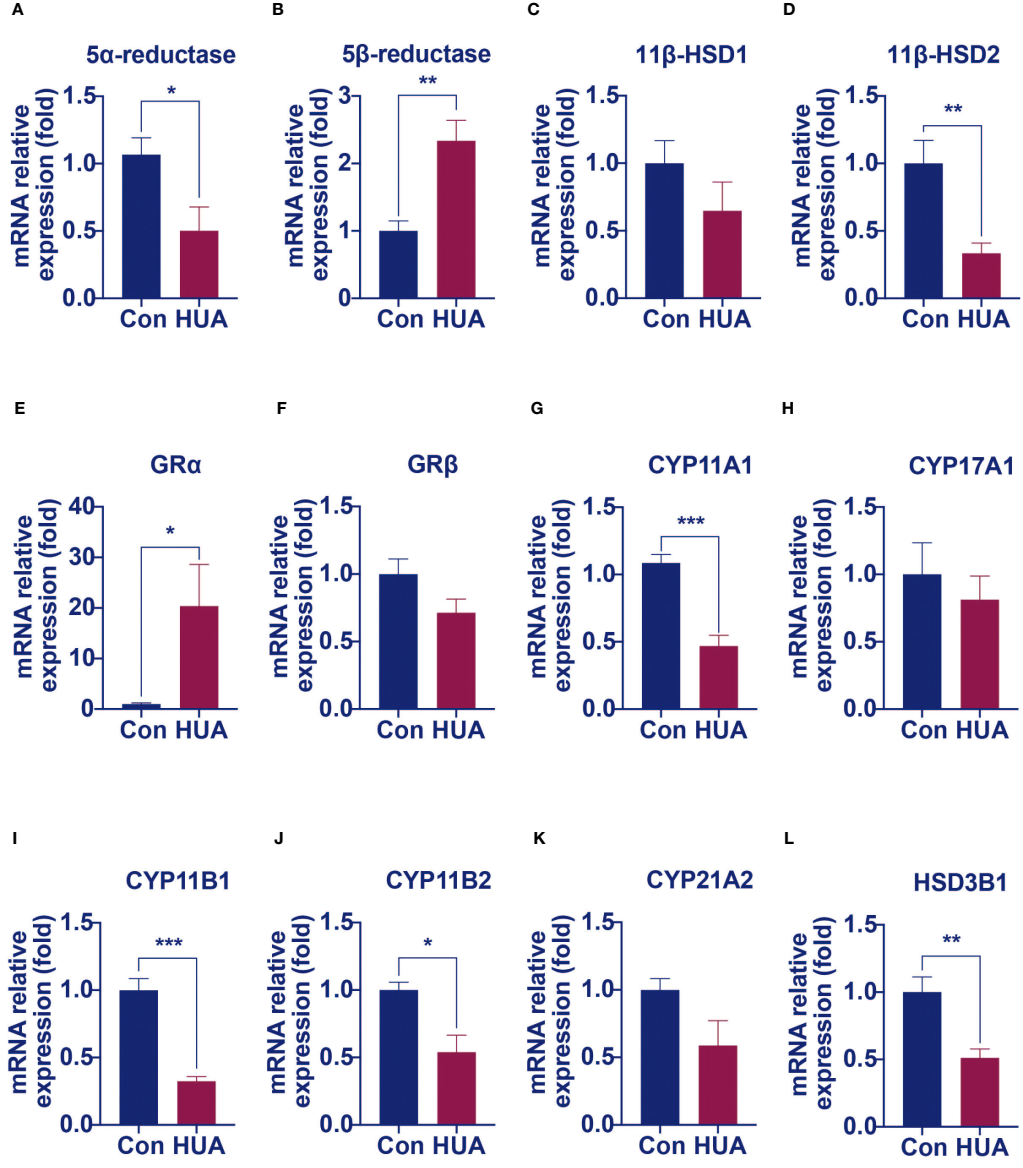
The mRNA levels of genes involved in cortisol clearance and steroidogenesis decreased in hyperuricemia mice. Normal control C57BL/6J mice group (Con), PO and adenine induced hyperuricemia mice group (HUA). **(A, B)** The hepatic mRNA levels of 5α-reductase and 5β-reductase. **(C, D)** The mRNA levels of genes involved in regenerating cortisol from inactive cortisone. **(E, F)** The hepatic mRNA levels of glucocorticoid receptor α and β. **(G–L)** The mRNA levels of steroidogenesis‐related genes in mouse adrenals. Data are presented as mean ± S.E.M. ^*^ *p* < 0.05, ^**^ *p* < 0.01, ^***^ *p* < 0.001 vs control mice group.

We hypothesized that damage of the liver cells impaired the clearance of cortisol and reduced cortisol production by suppressing the HPA axis. It is well known that the principal cortisol clearance occurs in the liver (5α, β-reductase) and the kidney (11β-hydroxysteroid dehydrogenase 2, 11β-HSD2), through which cortisol is eventually converted to cortisone, and excreted in urine (10, 11). Compared to the control, the hyperuricemia mice had a reduced mRNA level of hepatic 5α-reductase (**Figure 5A**) and increased hepatic 5β-reductase (**Figure 5B**), with no difference in the mRNA level of hepatic 11β-HSD1 (**Figure 5C**). In the kidney, 11β-HSD2 expression was down-regulated by 66% compared to the control (**Figure 5D**). It was reported that 5α-reductase plays a more important role than 5β-reductase in cortisol clearance (11). The above results indicated that cortisol clearance was impaired in a hyperuricemia state. Further gene expression studies revealed that glucocorticoid receptor α (GRα), but not GRβ was significantly up-regulated in the liver of the hyperuricemia mice, which confirmed the increased exposure of cortisol in the liver (**Figures 5E, F**).

Cortisol synthesis is a tightly controlled pathway, with over 10 enzymes involved. We next examined the mRNA levels of these enzymes. Compared with the control mice, the mRNA levels of enzymes such as cytochrome P450 family 11 Subfamily a member 1 (CYP11A1), 11β-hydroxylase (CYP11B1), aldosterone synthase (CYP11B2) and 3β-hydroxysteroid dehydrogenase 1 (HSD3B1) were significantly lower in the adrenal of hyperuricemia mice. Other enzymes, such as cytochrome P450 family 17 subfamily A member 1 (CYP17A1) and 21-hydroxylase (CYP21A1), showed no significant changes under hyperuricemia conditions (**Figures 5G–L**). These results were consistent with our previous findings of lower total cortisol levels in the hyperuricemia mice.

## 4 Discussion

Long-term high serum uric acid level is a risk factor of hyperlipidemia, and cardiovascular disease. However, the underlying mechanism is unclear. By analyzing hyperuricemia patients’ blood samples, we found that approximately half of the hyperuricemia patients had elevated GGT levels, indicating that hyperuricemia caused hepatic injury and mild cholestasis. To date, the mechanism of this pathophysiological process has not yet been clarified.

Both imbalanced cortisol metabolism and extrinsic glucocorticoids can cause hepatic injury, dysmetabolism of cholesterols, depression, and hypertension etc. (12–14). It had been reported that abnormal cortisol metabolism induced cholestatic liver injury (6). In the cholestasis mice with single-sided adrenalectomy, the hepatic injury was alleviated along with decreased serum glucocorticoid levels. Meanwhile, the expression of HMGCR was reduced, and serum cholesterol levels declined. Similar effect was observed when using glucocorticoid receptor antagonists (6).

In this study, we discovered the hyperuricemia induced dysmetabolism of cortisol for the first time. Based on the clinical and experimental data, we found that hyperuricemia was associated with lower total cortisol levels, higher urine free cortisol levels, and slower cortisol clearance when compared with the controls. We designated this condition as “PHAL”.

Low total cortisol level is usually due to decreased adrenal production. We found in this study that in PHAL, the ACTH levels were within normal limit. However, the adrenal glands failed to respond to ACTH properly, leading to low cortisol production, and decreased expression levels of CYP11A1, CYP11B1/2, and HSD3B1, three key enzymes for cortisol synthesis.

The degradation of corticosteroids starts as soon as the hormones are released into blood. The amount of bioavailable cortisol is determined by the hepatic and renal clearance (15). The steroid 5α and 5β-reductases in the liver metabolize cortisol to tetrahydrocortisone, where the 5α-reductase is the major enzyme. The 11β-HSD2 in the kidneys converts cortisol to cortisone (10). In this study, we found that hyperuricemia significantly reduced the expression of both hepatic 5α-reductase and renal 11β-HSD2, which led to low cortisol clearance.

More than 90% of the metabolites of corticosteroid hormones are excreted in urine. In PHAL, the excretion of corticosteroids through the kidneys was 70% higher than that under normal condition, further indicating that low hepatic and renal clearance led to more undegraded free cortisol excreted in urine.

Based on the above results, we postulated a possible pathogenic mechanism of PHAL induced cholesterol metabolism disorder under hyperuricemia conditions. PHAL increases exposure to the bioavailable cortisol in organs such as the liver, leading to local amplification of the biological action of corticosteroids. This causes over-activation of the corticosteroid receptors locally. If this condition persists, it may lead to inhibition of the HPA axis via negative feedback, downregulate the production of cortisol compensatorily, and cause adrenal atrophy, which further exacerbates cortisol dysmetabolism.

PHAL is different from Cushing’s syndrome and adrenal insufficiency (**Table 4**). In Cushing’s syndrome, both serum cortisol and ACTH levels are higher than normal, which is not suppressed by low dose dexamethasone. Urine cortisol levels are high in Cushing’s syndrome. In PHAL, total cortisol levels are lower than normal, with ACTH in the normal range. Low dose dexamethasone only suppresses ACTH, but does not reduce cortisol levels. Urine cortisol levels are also high in PHAL. In adrenal insufficiency, total cortisol levels are low, with high or low ACTH levels in primary or secondary adrenal insufficiency, respectively. Urine cortisol levels are usually low in adrenal insufficiency. In addition, relative adrenal insufficiency had been observed in other chronic disease, such as cholestasis (insufficient in cortisol clearance) (31, 32), and functional hypercortisolism in metabolic syndrome (11β-HSD1 and the pre-receptor regulation) (33, 34). Serum cortisol level was either increased or normal in relative adrenal insufficiency, different from that in hyperuricemia induced PHAL. Intestinal and hepatic FXR were commonly suppressed in cholestasis, and contrarily activated in PHAL. This difference may come from metabolites of purine, which induced hepatic cell injury from different pathological way. The mechanisms need to be further investigated.

**Table 4:** Altered cortisol in Pseudohypoadrenalism, Cushing’s syndrome and Adrenal insufficiency.

	Pseudohypoadrenalism	Cushing’s syndrome	Adrenal insufficiency
Serum cortisol	Low	Normal or high (16)	Low (17)
24h total Urine cortisol	High	High (18)	Low (17)
Cortisol rhythm	Normal	Failure to achieve a normal late-night circadian nadir (19–22)	The amplitude of the cortisol rhythms is dampened (23)
HPA axis	Suppression	Impairment of the normal feedback of the HPA-axis (16)	Suppression (24)
liver	5α reductase decreased 5β reductase increased GRα increased GRβ normal 11β HSD1 normal	5α reductase decreased 5β reductase normal GR increased (25)	5α reductase decreased (11)
Kidney	11β HSD2 decreased	11β HSD2 decreased (26)	11β HSD2 decreased (27)
Adrenal	CYP11A1 decreased CYP17A1 normal	CYP11A1 increased (28, 29)	CYP11A1 insufficiency (30) 11β-hydroxylase deficiency 17α-hydroxylase deficiency (24)
ACTH	Normal	ACTH-dependent Cushing’s syndrome (Cushing’s disease: normal; Ectopic ACTH: high) ACTH-independent Cushing’s syndrome (Adrenal tumor): low (26)	Primary adrenal insufficiency: high Secondary adrenal insufficiency: low (24)

Our group had focused on the effect of PHAL on metabolic diseases. Imbalanced corticosteroid hormones are known to cause not only hypercholesterolemia, but also hyperlipidemia, non-alcoholic fatty liver disease, cardiovascular diseases, and diabetes (35, 14). These metabolic diseases are common comorbidities in a large percentage of people with hyperuricemia. In the future, we will further our work on the relationship between PHAL and metabolic diseases.

In summary, cortisol is involved in hyperuricemia associated dysmetabolism of cholesterols. Clinically, attention should be paid to the cortisol levels while managing hyperuricemia. PHAL is a mild form of cortisol dysmetabolism, with nearly intact adrenal function. Hormone replacement is not necessary for PHAL. Maintaining homeostasis of cortisol metabolism in PHAL could be achieved by adjusting circadian rhythm, exercise, or amino acid supplementation, in order to subsequently ameliorate dysmetabolism of cholesterols related to hyperuricemia. More studies are needed to further clarify the mechanism of PHAL, which may offer new therapeutic strategies for hyperuricemia and its complications.

## Data availability statement

The original contributions presented in the study are included in the article/supplementary material. Further inquiries can be directed to the corresponding authors.

## Ethics statement

The studies involving humans were approved by the Second teaching hospital of Tianjin University of Traditional Chinese Medicine. The studies were conducted in accordance with the local legislation and institutional requirements. The participants provided their written informed consent to participate in this study. The animal study was approved by the Science and Technological Committee and the Animal Use and Care Committee of TJUTCM. The study was conducted in accordance with the local legislation and institutional requirements.

## Author contributions

RB: Conceptualization, Methodology, Writing — original draft, Investigation. BC: Formal analysis, Methodology, Writing — original draft. JP: Investigation, Methodology, Writing- review & editing. AW: Investigation, Writing — review & editing. HY: Data curation, Formal analysis, Writing — review & editing. QC: Data curation, Formal analysis, Writing — review & editing. YZ: Conceptualization, Investigation, Writing — review & editing. TW: Conceptualization, Project administration, Resources, Writing — review & editing.
